# Supplementary figures and images for: Neuregulin-1 prevents death from a normally lethal respiratory viral infection
Source: PLoS Pathog. 2025 Apr 23;21(4):e1013124. doi: 10.1371/journal.ppat.1013124 (PMC12052188; doi:10.1371/journal.ppat.1013124)

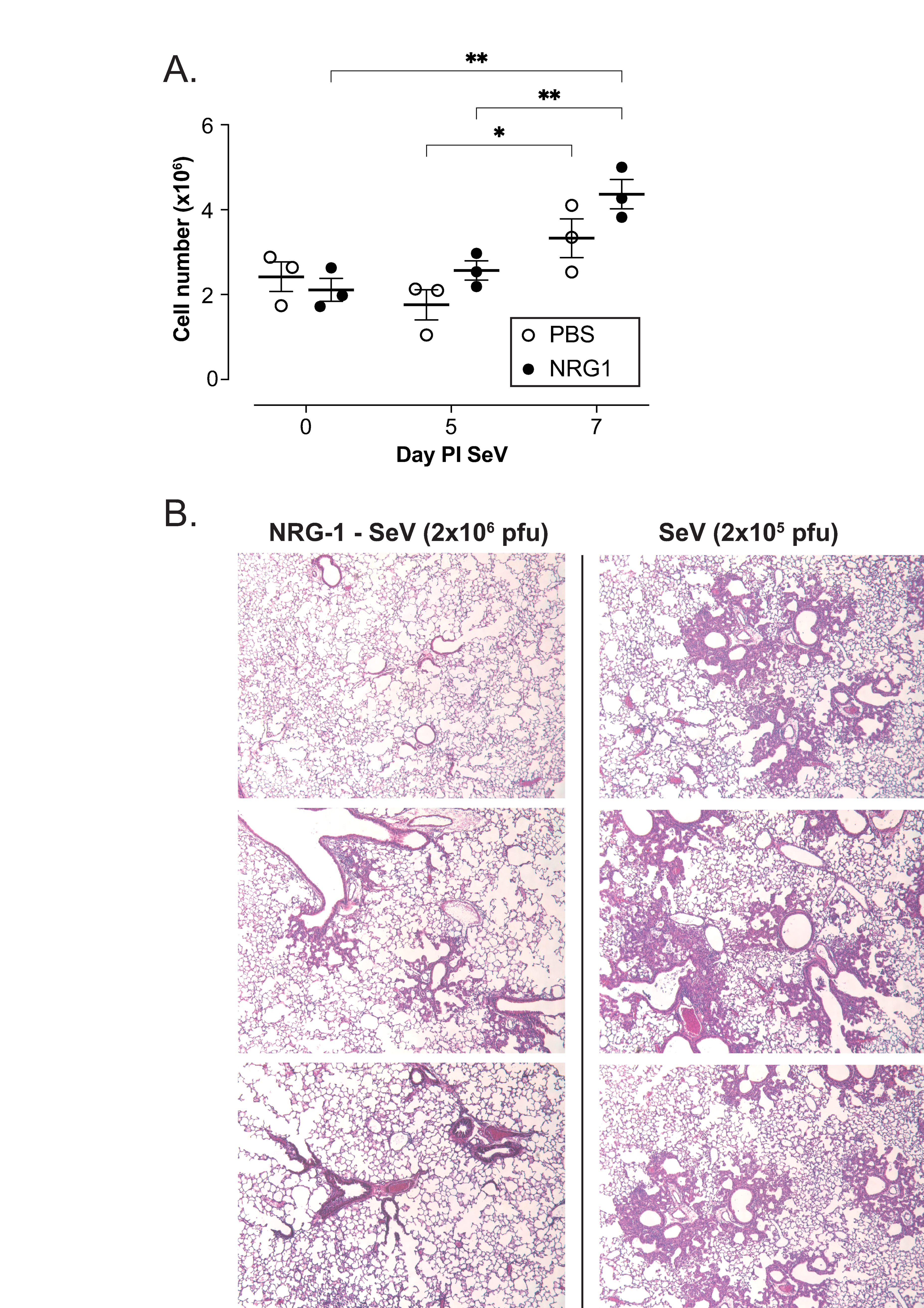

Supplement: S1 Fig — (A) Granulocyte numbers determined from flow cytometric analysis based on forward and side scatter demonstrating no significant difference in high dose SeV infected mice treated with PBS or NRG1 as in Fig 2A at d0, d5, d7 PI SeV (n = 3). (B) Hematoxylin and eosin (H&E) staining of paraffin fixed mouse lung comparing high dose SeV (2x10^6^ pfu) infected and NRG1 treated (NRG-1-SeV) mice with regular dose SeV (2x10^5^ pfu) infected but otherwise untreated mice at day 21 post infection showing markedly reduced inflammation in the NRG1 treated group even with the higher viral dose. Images taken at 5x magnification, n = 3 per group. (TIF) [file ppat.1013124.s002.tif]

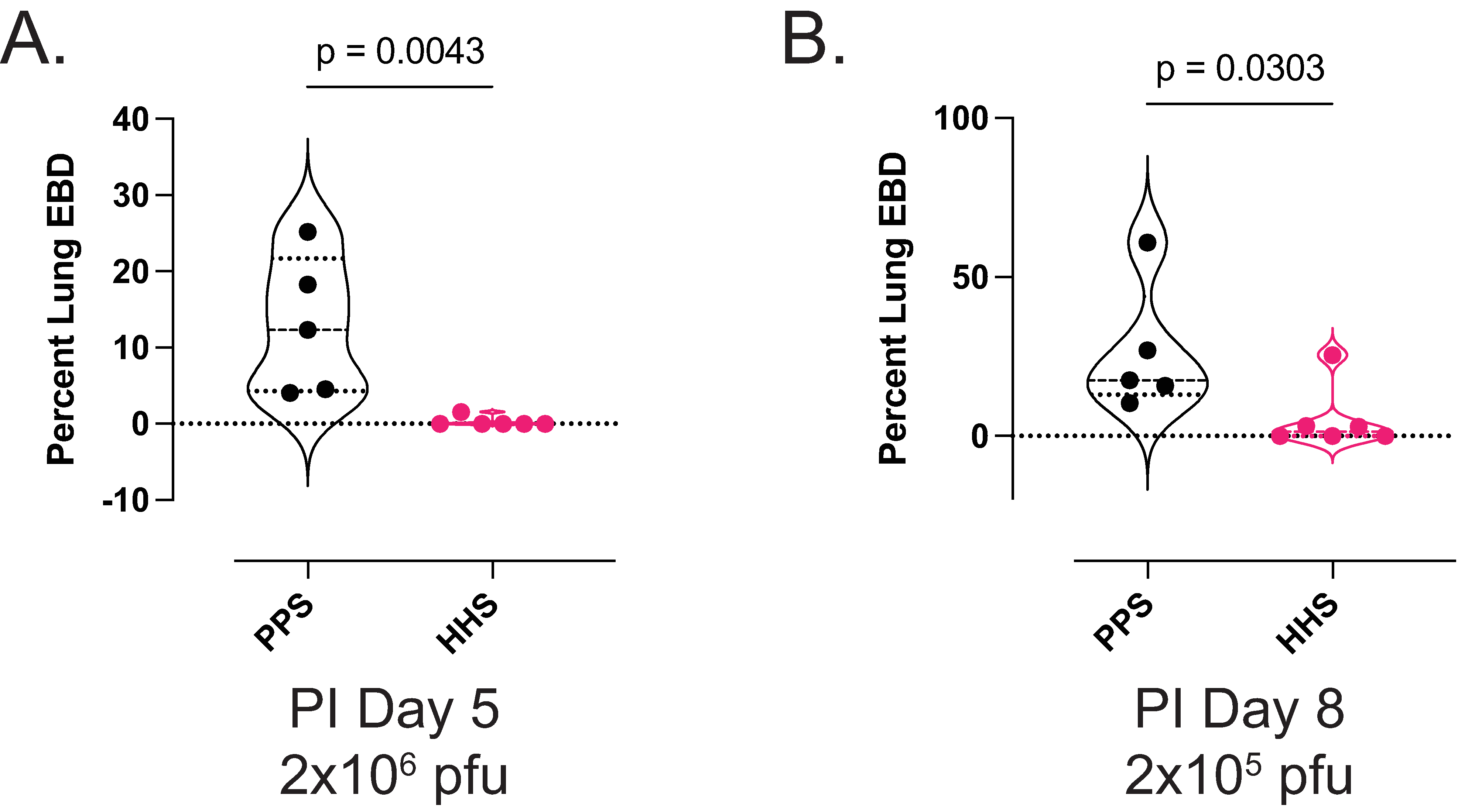

Supplement: S2 Fig — (A) Ratio of EBD in the airway (BAL) to lung at day 5 post inoculation (PI) high dose SeV (2x10^6^ pfu) is lower for mice made atopic (HHS, red circles) compared to non-atopic mice (PPS, black circles). (B) Similar to (A) but using regular dose SeV (2x10^5^ pfu) and measuring EBD at day 8 PI. For (A) and (B) median ± IQR shown, Mann-Whitney U test, n ≥ 5. (TIF) [file ppat.1013124.s003.tif]

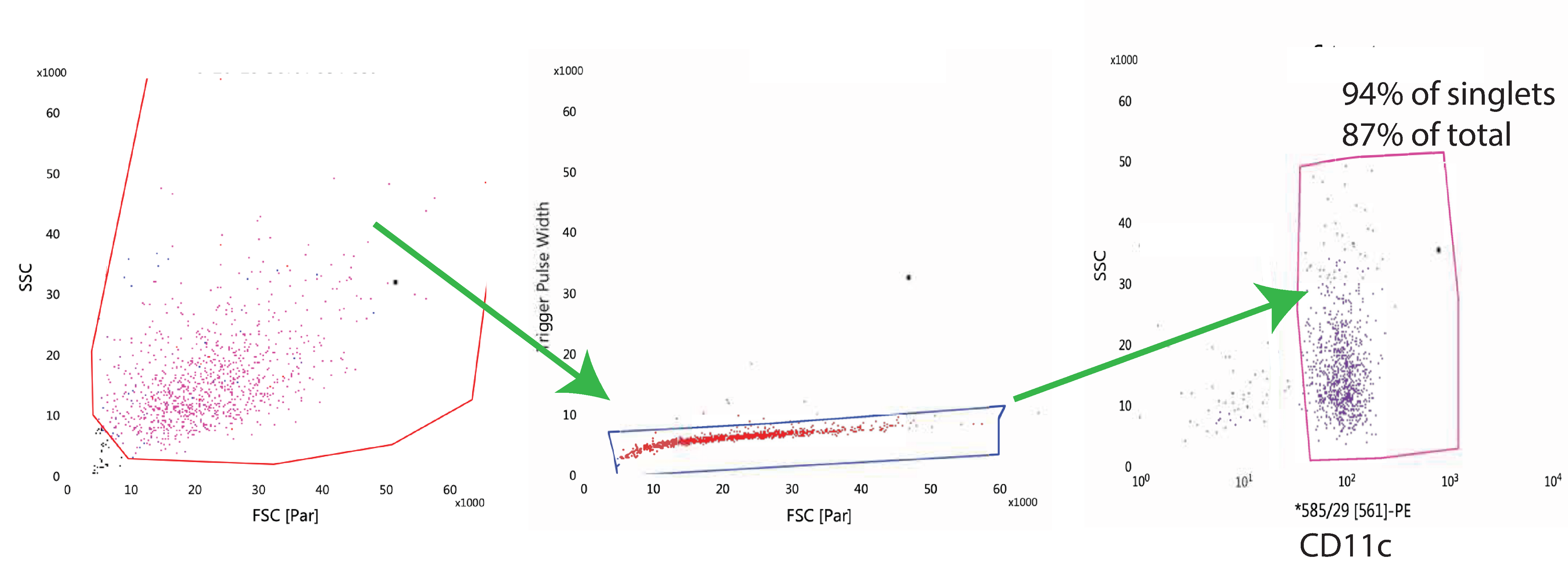

Supplement: S3 Fig — FACS of CD11c+ cells from lung stained with PE conjugated CD11c antibody shows gating strategy which led to ≥ 87% purity of the selected population (left panel: FSC vs SSC, middle panel: singlets, right panel: CD11c+ population). (TIF) [file ppat.1013124.s004.tif]
