# Supplementary material for: Neuregulin-1 prevents death from a normally lethal respiratory viral infection
Source: PLoS Pathog. 2025 Apr 23;21(4):e1013124. doi: 10.1371/journal.ppat.1013124 (PMC12052188; doi:10.1371/journal.ppat.1013124)
Supplement: Attachment — Submitted filename: Response to Reviewers Plos Path 02032025.pdf [file ppat.1013124.s006.pdf]

PPATHOGENS-D-24-02280
Neuregulin-1 inhibits mouse respiratory virus induced necroptosis and death
PLOS Pathogens

Response to Reviewers

Additional Editor Comments:

This manuscript has been reviewed by three experts. As a result, some reviewers pointed out that the association between the expression level of NRG1 and its effect on mitigating disease severity has not been sufficiently demonstrated. Additionally, one reviewer has raised questions regarding the novelty of the study. On the other hand, another reviewer acknowledged the novelty of this research and evaluated it as a significant contribution to the field. Please carefully consider all reviewers' comments and provide thorough responses accordingly.

Reply: We thank the editors and reviewers for their careful evaluation of our manuscript. In the revision we have provided additional detail on the effect of NRG1 in mitigating the respiratory viral infection (see our responses to the reviewers for details). As mentioned below, the novelty in our manuscript is the role of NRG1, and we do not claim that NRG1 is the mechanism of protection in atopic mice, just that we identified NRG1 from atopic mice. We hope this clarifies the novelty of our report and that it is now acceptable for publication in PLOS Pathogens.

Journal Requirements:

We are only including the points in Journal requirements that required specific responses from us.

4) We noticed that you used the phrase 'not shown' in the manuscript. We do not allow these references, as the PLOS data access policy requires that all data be either published with the manuscript or made available in a publicly accessible database. Please amend the supplementary material to include the referenced data or remove the references.

Reply: We have removed the ‘not shown’ phrase and have included these data in supplemental figure S3 (purity of FACS sorted CD11c+ cells).

5) We do not publish any copyright or trademark symbols that usually accompany proprietary names, eg ©, ®, or TM (e.g. next to drug or reagent names). Therefore, please remove all instances of trademark/copyright symbols throughout the text, including:

- ® on page: 18
- TM on pages: 18, 21, and 23.

Reply: Proprietary names with trademark symbols have been removed from all reagents/material used.

7) Please upload a copy of Figure 4E which you refer to in your text on page 12. Or, if the figure is no longer to be included as part of the submission please remove all reference to it within the text.

Reply: Thank you for pointing out this oversight. We mistakenly wrote the incorrect figure number in the text and have now corrected this error (the correct figure is 6E).

8) Please label the table and amend its reference accordingly.

Reply: We have amended the table reference and provided a label, as requested.

9) We have noticed that there is a reference to supplemental figure 2 on page 19. However, there is no corresponding file uploaded to the submission. Please upload it as a separate file with the item type 'Supporting Information'.

Reply: We apologize for this error. This information was actually provided in figure 5B (not in a supplemental figure) however, in the revised manuscript we have rearranged Figs 3-5 so now the Fig 5B is Fig 4D and we have amended the text to reflect this fact.

10) We have noticed that you have uploaded Supporting Information files, but you have not included a list of legends. Please add a full list of legends for your Supporting Information files after the references list.

Reply: The supporting file was an Excel file showing differential gene expression of RNA seq samples. We have added a legend in the manuscript after the references list.

11) Thank you for stating that " I have deposited our RNA seq data, relevant to the submitted manuscript (PPATHOGENS-D-24-02280) to GEO repository following is the link and accession no

https://www.ncbi.nlm.nih.gov/geo/query/acc.cgi

GEO accession: GSE281143" . We noticed that the data is currently private and is scheduled to be released on Nov 04, 2025. We strongly recommend all authors deposit their data before acceptance, as the process can be lengthy and hold up publication timelines. Please note that, though access restrictions are acceptable now, your entire minimal dataset will need to be made freely accessible if your manuscript is accepted for publication. This policy applies to all data except where public deposition would breach compliance with the protocol approved by your research ethics board. If you are

unable to adhere to our open data policy, please kindly revise your statement to explain your reasoning and we will seek the editor's input on an exemption.

Reply: We agree with this policy and will make sure that the entire minimal dataset is made freely accessible (i.e., the access restrictions are fully lifted) upon notice that our manuscript is accepted for publication.

However, the following secure token has been created to allow review of record GSE281143 while it remains in private status:

To review GEO accession GSE281143:
Go to
https://urldefense.com/v3/__https://www.ncbi.nlm.nih.gov/geo/query/acc.cgi?acc=GSE281143__;!!NiUAmZJ8c1GNWg!XsKaBHcJ35y3J7LCMPB7qUOy8wbrCybSfPd1oUTLA5TM8qaHZvIr_JS6lk658_GwLHbNTOgwLpbX7Rd75oMTxPmtaLjJa_U$
Enter token urgxoekchpwtvkf into the box

Reviewers' Comments:

Reviewer's Responses to Questions

Part I - Summary
Please use this section to discuss strengths/weaknesses of study, novelty/significance, general execution and scholarship.

Reviewer #1: 1. Lack of Novelty: The findings on the protective effect of atopy in viral infections lack novelty, as similar protective mechanisms have been demonstrated in previous studies using influenza virus in mouse models. Studies have already shown that pre-existing atopy can reduce disease severity in influenza infections through mechanisms such as NK cell activation and Type III interferon induction

Reply: We agree with the reviewer that protection against viral infection due to pre-existing atopy has been reported previously. We have also provided a mechanistic explanation for the inhibition of post-viral airway disease in atopic mouse model (Hussain et al., J Immunol. 2021;207(10):2589-97). However, the focus of our current study is on neuregulin-1 (NRG1), which is highly expressed in atopic mice and is considered as one of the regulators of goblet cell formation in human airway epithelial cells. We utilized the atopic model only in that we looked for differentially expressed gene products between atopic and non-atopic mice and found NRG1. The novelty of our study, as it is acknowledged by reviewer #2, is to show that NRG1 on its own in non-atopic mice is sufficient to provide a survival advantage against a lethal dose of both IAV and Sendai virus (a rodent RNA virus). To our knowledge this is the first study to demonstrate that NRG1 (or any ErbB receptor agonist) has a protective role in a

respiratory viral infection. However, as we have stated in the manuscript, we do not claim that NRG1 is the mechanism of survival in atopic mice and further studies will be needed to demonstrate if NRG1 is sufficient and/or required for the protection we see in atopic mice.

2. The authors challenged C57BL6 mice intranasally with 10 μg of house dust mite (HDM) extract 3 days before SeV infection, but they have not ruled out the possibility that the HDM extract could induce nonspecific innate immunity, including interferons, which may improve the survival rate of virus-infected mice or suppress SeV replication.

Reply: We thank and agree with the reviewer for pointing out that innate immunity as potential reason for survival in atopic mice. However, as stated above our focus was to understand how NRG1 on its own is able to provide protection from viral infection; the atopic model was only the means by which we identified NRG1. We do not dispute the possibility that there are several mechanisms that could lead to survival in atopic mice (see response to query #1), but in this study we are focusing on the effect of NRG1 in non-atopic mice. In terms of the mechanism of NRG1 mediated protection, we did examine the expression of genes associated with homeostatic/protective function of airway epithelium (see table1 and Fig 7) but did not specifically look at the expression of interferons.

3. Rational: Despite the RNAseq analysis results showing significant differences in the expression levels of various genes, including Adam11, between NA mice and atopic mice, the rationale for focusing on neuregulin-1 is insufficient.

Reply: The reviewer has raised a valid question for the clarification of our rationale in choosing NRG1 and not the other more highly expressed genes such as Adam11 in atopic mice. The reason we selected NRG1 to study the effect on respiratory viral infection was based upon multiple factors -- first this protein plays a major role in epithelial homeostasis and regeneration following damage. Also, some studies have shown it to have anti-fibrotic effects in the lungs. Though studies have shown beneficial effects on the lung, no study (until ours) has shown a role for NRG1 in providing a survival advantage against respiratory viral infections.

4. Limited Mechanistic Insight: The manuscript does not provide sufficient mechanistic insight into how neuregulin-1 (NRG1) mediates protection against respiratory viral infections. While the authors highlight NRG1's role in reducing necroptosis, they do not adequately demonstrate how this leads to the observed survival benefit, leaving gaps in the mechanistic understanding.

Reply: We agree with the reviewer that an in-depth mechanistic analysis is warranted to fully understand the signaling pathways and cellular interactions that may be involved in NRG1 mediated protection against respiratory viral infection. In fact, this underlies a

major issue in the field, as the actual mechanism of lethality from a respiratory viral infection is not well understood. During the preparation of our manuscript a study was published demonstrating that direct necroptosis inhibition provides survival benefit against viral infections (Gautam et. al., *Nature*, 2024, vol 628, 835-842, doi.org/10.1038/s41586-024-07265-8). In our study, NRG1 treatment reduced not only necroptosis but also airway epithelial leak both *in vitro* and *in vivo*. We have revised the manuscript to elaborate upon the potential mechanisms of survival due to necroptosis inhibition.

5. Overemphasis on Correlative Data: Much of the evidence provided in the study appears to be correlational, particularly regarding the association between reduced necroptosis and improved survival. Stronger causal data, such as using knockout models, could strengthen the conclusions.

Reply: Kindly see our response to #4 regarding the reason for necroptosis inhibition providing improved survival. Regarding the suggestion to use knockout models for causality, there is no global *Nrg1* knockout model available (*Nrg1* deletion is embryonically lethal). We could use mice deficient in *Ripk3* or *Mlkl* to demonstrate the importance of necroptosis in the lethality to a respiratory viral infection. However, this has been done (see the Nature reference mentioned in our response to query 4), and therefore we do not think it would add anything further to our studies.

Reviewer #2: In this manuscript, Hussain SA et al. demonstrated the effect of pre-existing atopy on respiratory virus infection. The authors found that increased NRG1 protects mice from SeV infection via upregulation of repair process. This manuscript presents novel findings, but the results appear somewhat disconnected and lack a cohesive conclusion.

Reply: We thank the reviewer for acknowledging the novelty of our studies and providing valuable critique on the conclusion. We have now rephrased the conclusion paragraph to make it more cohesive.

Reviewer #3: Following up on previous work by the authors describing reduced severity of respiratory viral infections in mice made atopic, this study reports upregulation of NRG1 in atopic mice. In a series of in vivo and in vitro experiments, NRG1 is shown to mediate increased survival of animals in a SeV mouse infection model through reduced alveolar leakage and inhibition of virus-induced necroptosis.

These results advance the mechanistic understanding of an unexpected phenotype and identify determinants of disease severity. Data are well presented and overall support the conclusions drawn. Although no direct causality between NRG1 expression levels and greater protection of atopic mice has been established, the authors recognize and appropriately discuss possible limitations of the work. Thorough final editing of the text is recommended to address a few inaccuracies (i.e. reporter gene expression is not

equivalent to virus titer (line 256)), but overall this is an important study that will advance the field.

Reply: We thank the reviewer for their kind comments and appreciate that they believe this is a significant contribution to the field. We acknowledge we have not established a direct link between Atopy-NRG1-Survival. However, as noted in our responses to reviewer 1 comments 1 & 2 our focus was on NRG1 alone, not NRG1 in mediating atopic protection.

We also want to thank the reviewer for pointing out that reporter gene intensity should not be termed as equivalent to viral titer and have corrected this in the revised manuscript.

Part II – Major Issues: Key Experiments Required for Acceptance
Please use this section to detail the key new experiments or modifications of existing experiments that should be absolutely required to validate study conclusions.

Generally, there should be no more than 3 such required experiments or major modifications for a "Major Revision" recommendation. If more than 3 experiments are necessary to validate the study conclusions, then you are encouraged to recommend "Reject".

Reviewer #1: (No Response)

Reviewer #2: 1. In line 87: “our high-fidelity model of respiratory viral infection utilizes a natural rodent pathogen, SeV…”, SeV infection cannot be an animal model for RSV infection in humans. It is better to reorganize this section.

Reply: Thank you for pointing out and suggesting reorganizing the section. We have now changed the wording to indicate that Sendai virus is a negative strand respiratory RNA virus and causes some of the similar cardinal feature of RSV, IAV and PIV in mice.

2. Fig 1B: please provide MLD50 of SeV infection. How much mice are survived from the “regular” dose infection?

Reply: We have not calculated the exact MLD50 for SeV; however, as reported in the literature, mortality of up to 20% is seen with the $2x10^5$ pfu dose (Nat Med 2005;11:1180–1187). We do not exactly know how this correlates to gSeV copy number, but based on the data in Fig 1B, we would assume it is likely somewhere in the

1-3x10^6 gSeV copies. This would correlate with the atopic mice having a much higher copy number than would be associated with complete survival. We have added this mortality information to the text (in the introduction when we first discuss SeV titers).

3. The authors need to show the severity of asthma in this model. In this model, there is no PAS+ cells as shown in Fig 2E, indicating that the asthma symptoms are very weak. Please examine levels of IgE and granulocytes. Is this a chronic inflammation model?

Reply: As pointed out by the reviewer there are limited number of PAS+ cells in the regular SeV infected mice; however, this is in-line with our previously published report (J Immunol 2021;207(10):2589-2597). Nonetheless, as shown in that prior study, there was still significant airway hyperreactivity (in the absence of pre-existing atopy). The HDM model we used for the studies can be temporally described as an acute model as we only challenged with HDM for 5 days after a single sensitization, unlike the chronic model where HDM is given 5 days/week for five weeks. In this model, as we have reported earlier (*J Immunol* 2021;207(10):2589-2597), there is an early cellular infiltration and inflammatory phase which is resolved by day 14. However, as we have responded to reviewer 1's comments 1 & 2 the focus of our study was to understand how NRG1 is preventing death from respiratory viral infection, therefore, we have now examined granulocyte levels in NRG1 treated lungs at day 0, and days 5 & 7 post infection with high dose SeV. Although the granulocyte number increases with infection there was no significant difference between PBS+SeV group and the NRG1+SeV group on any of the days examined. Further, H & E staining of lungs at day 21 post infection was performed in NRG1 treated mice that received the high dose of SeV and non-atopic mice given the regular dose of SeV (since high dose SeV treated non-atopic mice would not live to day 21). Interestingly, the NRG1 treated mice have almost no inflammation in their lungs (even though they received the higher dose of SeV) compared to the continued level of inflammation in the regular dose SeV infected mice. We have now included these results in supplemental information (Fig S1) and incorporated additional discussion in the manuscript.

4. In line 161: "In a dose responsive fashion", there is no dose response.

Reply: Thank you for pointing out the inaccuracy in wordings to show the effect of various NRG1 concentrations used. This has now been corrected in the manuscript.

5. Fig 3: EBD should be examined in SeV-infected atopic model.

Reply: We have now included EBD for SeV-infected atopic mice as supplemental information (Fig S2). The EBD data in atopic mice demonstrates reduced EBD in the BAL similar to what we see in NRG1 treated non-atopic mice.

6. Fig 2C: The viral titer does not drop in NRG1 treated mice, even though it does in the atopic model.

Reply: Thank you for raising the issue of viral titer. Although we don't have a clear understanding why intranasal NRG1 does not affect viral titer, it still provides protection against mortality. We believe the mechanism of survival is independent of viral titer. Indeed, with viruses such as RSV, viral titer does not always corelate with disease severity (Walsh et. al., J Infect Dis . (2018) doi: 10.1093/infdis/jiy106). Moreover, our *in vitro* data demonstrate that when NRG1 is applied to the apical side of the epithelial cells, airway leak is completely prevented unlike when NRG1 is presented basally; however, apical administration of NRG1 led to less of a reduction in viral replication than when it was applied basally, suggesting a disconnect between viral titer and airway leak (See Fig 6 C& D). Further, in atopic mice, while viral titer is reduced, it remains significantly higher than that seen in regular dose SeV infected non-atopic mice (Fig 1B).

7. Fig 5A: The results are very unclear, the authors have to provide WB results with similar lysate amount.

Reply: We apologize if this was not clear in our original report, but using a Bradford assay we loaded equal amounts of lysate protein in each lane (50 mcg) of the gel. The data analysis in the WB was based upon the normalized intensity of target proteins (MLKL or pMLKL) to the loading control (GAPDH or beta-Tubulin) within the same lane, which compensates for variation in loading amount. As such, we have not changed the original figure (note: since we have rearranged figures 3-5 now the western blot is now Fig 4C, D).

8. It is unclear why necroptosis is inhibited by NRG1 treatment.

Reply: Please see our response to reviewer 1's Part 1 comments #4 and #5. Furthermore, NRG1 is known to activate the AKT signaling pathway which in turn can inhibit RIPK3/ MLKL leading to inhibition of necroptosis (Laskin et al., Ann Oncol (2022), doi: 10.1016/j.annonc.2020.08.233, Zhu et al., *Burns & Trauma (2024)* https://doi.org/10.1093/burnst/tkae035). Although we acknowledge that we have not studied AKT signaling in our model, we did look at the final necroptosis protein MLKL and found that it is less activated in the NRG1 treated group compared to mice infected with SeV alone.

9. Is the epithelial leak regulated by necroptosis?

Reply: We have used a known necroptosis inhibitor, necrosulfonamide (NSA) in human hBEC with RSV infection and shown a reduction in epithelial leak (Fig 6E). Therefore, it is a strong possibility that necroptosis directly regulates epithelial leak. Since NSA has been reported to be ineffective in mice (Wan et al., J Natl Cancer Ctr (2022) https://doi.org/10.1016/j.jncc.2022.08.007), we are not able to show if inhibiting necroptosis would prevent epithelial leak *in vivo*.

Reviewer #3: (No Response)

Part III – Minor Issues: Editorial and Data Presentation Modifications
Please use this section for editorial suggestions as well as relatively minor modifications of existing data that would enhance clarity.

Reviewer #1: (No Response)

Reviewer #2: (No Response)

Reviewer #3: (No Response)
