# Supplementary material for: Neuregulin-1 prevents death from a normally lethal respiratory viral infection
Source: PLoS Pathog. 2025 Apr 23;21(4):e1013124. doi: 10.1371/journal.ppat.1013124 (PMC12052188; doi:10.1371/journal.ppat.1013124)
Supplement: Attachment — Submitted filename: Response to Reviewers PloS Path 040125 .docx [file ppat.1013124.s007.docx]

PLOS Pathogens: Decision on your submission PPATHOGENS-D-24-02280R1 - [EMID:8b30da5dbc8e3993]

PPATHOGENS-D-24-02280R1
Neuregulin-1 inhibits mouse respiratory virus induced necroptosis and death
PLOS Pathogens

**Response to Reviewers**

**Dear Editor,**

We are submitting the revised manuscript and have addressed the points raised by the reviewers.

**Additional Editor Comments:**

According to the concerns one of the reviewers raised, it is unclear what pathological condition this model reflects. In other words, it is difficult to determine whether this model has aspects of an atopic model or whether the inflammation-inducing model using HDM is merely a trigger for discovering NRG1. Another particularly important point is whether the observed immune and inflammatory response, and pathological features are completely consistent with necroptosis. This is because the title of this manuscript is based on the claim that NRG1 inhibits necroptosis, so a satisfactory explanation of these points is required. Is it possible to respond appropriately to the reviewer's concern?

Reply: Please see below for our comments regarding the HDM model. We agree that without an in vivo necroptosis inhibitor, we cannot prove that NRG1’s effects are entirely through inhibition of necroptosis. As such, we have changed the title to remove the statement regarding necroptosis.

*New title:*

Neuregulin-1 prevents death from a normally lethal respiratory viral infection.

**Reviewers' Comments:**

Reviewer's Responses to Questions

**Part I - Summary**
Please use this section to discuss strengths/weaknesses of study, novelty/significance, general execution and scholarship.

Reviewer #1: (No Response)

Reviewer #2: Although HDM was administered, this is not an atopic model but rather an experiment in which mice were infected with artificially induced acute inflammation, not chronic inflammation, and there are discrepancies in the interpretation of the results. Necroptosis, which is an inflammatory cell death, was suppressed by NRG1, but inflammatory responses such as granulocyte recruitment were not suppressed. This suggests that necroptosis is not relevant to this mouse model. The results are not consistent and do not respond to this reviewer's concerns at all. Also, the data could not support the interpretation of the results.

Reply: We thank the reviewer for their comments and agree that NRG1 suppresses necroptosis, and this is associated with increased survival. This finding is the major point of our manuscript -- that NRG1 alone prevents mortality from normally lethal respiratory viral infections in naïve mice. The HDM atopic model was used initially just for our identification of NRG1.

Regarding the HDM administration, our HDM model is an acute atopic model as sensitization and challenge with HDM is done within 2 weeks unlike the chronic model where HDM is given 5 days/week for five weeks. However, this acute model of HDM has been reproducibly shown to elicit a strong cellular infiltration, serum IgE responses, mucus cell metaplasia (MCM) and airway hyperresponsiveness (AHR), sharing many similarities to human allergic asthma (Willart et al., *J Exp Med* 2012 Jul 30;209(8):1505-17); (Furuya et al., *Plos Pathogens* 2015, doi.org/10.1371/journal.ppat.1005180); (Hussain et al., *J Immunol* 2021;207(10):2589-2597); However, the early cellular infiltration and inflammatory phase is resolved shortly after the antigen is removed. Because we had previously shown that atopic mice do not develop post-viral airway disease at d21 post a non-lethal dose of SeV (Hussain et al., *J Immunol* 2021;207(10):2589-2597), we decided to determine if a similar situation occurred with the higher viral dose (the normally lethal dose in the atopic group), which it did – i.e., there was no increased AHR or MCM (fig 2D & E).

Regarding the effect of NRG1 and granulocyte numbers. We agree with the reviewer that in the first week of infection granulocyte numbers do not change in NRG1/SeV group compared to SeV alone. We assume this means that the effect of NRG1 is not through alteration of granulocytes. Indeed, as pointed out in our manuscript, we believe the primary effect (as is shown in the in vitro studies that do not include granulocytes) is due to alterations in epithelial cell responses to the viral infection (specifically reduced evidence of necroptosis). Regardless, by day 21 NRG1 leads to markedly reduced airway inflammation (by histology) even in mice given a normally lethal dose of SeV – even to levels that are lower than that seen in mice given the non-lethal SeV dose (but not treated with NRG1; supplemental fig 1). We do acknowledge that neutrophils (through NETs) can induce epithelial necroptosis and that in some models necroptosis increases neutrophil recruitment. However, in our study there is an ongoing respiratory viral infection, so recruitment of neutrophils is likely through numerous mechanisms. Further, the necroptosis seen in our study does not depend upon neutrophils, since we see necroptosis in vitro with virally infected hBECs (where there are no neutrophils).

As we have described and others have shown, NRG1 plays a role in maintaining the homeostasis of airway epithelium. Our data demonstrate that inflammatory cytokines such as IL-6 are reduced in NRG1 treated airway epithelium, along with a reduction in phosphorylated MLKL (a downstream target in necroptosis pathway). These data demonstrate NRG1 leads to a reduction in necroptosis which likely contributes to the survival. However, we cannot rule out other mechanisms of survival. We have added a statement to the manuscript regarding this possibility (lines 370-371), marked manuscript) and have changed the title to reflect this.

Reviewer #3: My concerns have been addressed. This is a well-executed study that will advance the field.

**Part II – Major Issues: Key Experiments Required for Acceptance**
Please use this section to detail the key new experiments or modifications of existing experiments that should be absolutely required to validate study conclusions.

Generally, there should be no more than 3 such required experiments or major modifications for a "Major Revision" recommendation. If more than 3 experiments are necessary to validate the study conclusions, then you are encouraged to recommend "Reject".

Reviewer #1: (No Response)

Reviewer #2: (No Response)

Reviewer #3: (No Response)

**Part III – Minor Issues: Editorial and Data Presentation Modifications**
Please use this section for editorial suggestions as well as relatively minor modifications of existing data that would enhance clarity.

Reviewer #1: (No Response)

Reviewer #2: (No Response)

Reviewer #3: (No Response)
